# Supplementary material for: Specialized impulse conduction pathway in the alligator heart
Source: eLife. 2018 Mar 22;7:e32120. doi: 10.7554/eLife.32120 (PMC5940360; doi:10.7554/eLife.32120)
Supplement: Supplementary file 1. — The presence of a specialized conduction system has been investigated in many reptile species and no consensus has emerged. The lack of consensus reflects the very heterogeneous quality of the previous studies and different definitions of ‘specialization’. To claim specialization, anatomists placed much emphasis on how pale cells were, that is how Purkinje cell-like they were, whereas many electrophysiologists placed emphasis on function, for instance whether there was an atrioventricular delay under the influence of nervous activity. Currently, ‘specialization’ is much informed by molecular biological data in the setting of the mammal heart. [file elife-32120-supp1.docx]

**Supplementary Table 1**. Methodologies and conclusions on the presence of a specialized conduction system in reptiles. (The numbers refer to References of Supplementary Table 1).

| Anatomy | Electrophysiology | Expression | Specialized - yes | Specialized - no |
| --- | --- | --- | --- | --- |
|  |  |  |  | 1,5 |
|  |  |  | 16,26 | 3,4,6,7,20,38,50 |
|  |  |  | 40,42,43 | 9,45,46 |
|  |  |  | 8,23,28,31,32,35 | 10,22,24,25,33,36,37,38,  39,41,44,47,48,49 |

The following studies on the reptile heart, could, but do not, concern the presence of specialized conduction system in reptiles ^2,11-15,17-19,21,27,29-30,34,45,50^.
